# Real-time diameter of the fetal aorta from ultrasound

**DOI:** 10.1007/s00521-019-04646-3

**Published:** 2019-12-18

**Authors:** Nicoló Savioli, Enrico Grisan, Silvia Visentin, Erich Cosmi, Giovanni Montana, Pablo Lamata

**Affiliations:** 1 Department of Biomedical Engineering, King’s College London, London, SE1 7EH UK; 2 Department of Computing, Imperial College London, Huxley Building, 180 Queen’s Gate, London, SW7 2RH UK; 3 Department of Woman and Child Health, University Hospital of Padova, Padova, Italy; 4 WMG International Digital Laboratory, University of Warwick, Coventry, CV32 7AL UK; 5 Department of Information Engineering, University of Padova, Padova, Italy

## Abstract

The automatic analysis of ultrasound sequences can substantially improve the efficiency of clinical diagnosis. This article presents an attempt to automate the challenging task of measuring the vascular diameter of the fetal abdominal aorta from ultrasound images. We propose a neural network architecture consisting of three blocks: a convolutional neural network (CNN) for the extraction of imaging features, a convolution gated recurrent unit (C-GRU) for exploiting the temporal redundancy of the signal, and a regularized loss function, called *CyclicLoss*, to impose our prior knowledge about the periodicity of the observed signal. The solution is investigated with a cohort of 25 ultrasound sequences acquired during the third-trimester pregnancy check, and with 1000 synthetic sequences. In the extraction of features, it is shown that a shallow CNN outperforms two other deep CNNs with both the real and synthetic cohorts, suggesting that echocardiographic features are optimally captured by a reduced number of CNN layers. The proposed architecture, working with the shallow CNN, reaches an accuracy substantially superior to previously reported methods, providing an average reduction of the mean squared error from 0.31 (state-of-the-art) to 0.09 $\mathrm{mm}^2$, and a relative error reduction from 8.1 to 5.3%. The mean execution speed of the proposed approach of 289 frames per second makes it suitable for real-time clinical use.

## Introduction

Fetal ultrasound (US) imaging plays a fundamental role in the monitoring of fetal growth during pregnancy and in the measurement of the fetus well-being. Growth monitoring is becoming increasingly important since there is epidemiological evidence that abnormal birth weight is associated with an increased predisposition to diseases related to cardiovascular risk (such as diabetes, obesity and hypertension) in young and adults [1, 2].

Atherosclerosis and cardiovascular disease in the adult population is linked to an increase of stiffness and thickness of major vessels. Similarly, accruing evidence suggests that the abnormal endothelization of major vessels during intra-uterine growth is linked to the same pathophysiological mechanisms as in adults, and even that fetuses born with endothelial damage have higher risks of adverse events in adulthood [3–6]. Intra-uterine growth restriction (IUGR) is the condition where these events are most prevalent, where fetuses experience an abnormal uterine environment either due to placental insufficiency or to nutrient/oxygen alteration.

The intima-media thickness (IMT) and the stiffness of the abdominal aorta by means of ultrasound examination are the most promising non-invasive biomarkers of adverse cardiovascular remodeling in fetuses and newborns [3, 6–12]. Fetal aortic IMT might be related to inflammation, probably indicating a very early stage of future atherosclerosis in adulthood [13]. The vision is that fetal aortic IMT and stiffness could become a decision marker of fetal damage in IUGR, i.e., to become a cardiovascular risk assessment biomarker complementing the weight percentile and the velocities (assessed by Doppler) of fetal vessels.

Obtaining reliable clinical metrics in IUGR is thus critically based on the accurate estimation of the diameter of the aorta over time. However, the poor signal to noise ratio of US data and the fetal movement makes the acquisition of a clear and stable US video challenging. Moreover, the measurements rely either on visual assessment at bedside during a patient examination, or on tedious, error-prone and operator-dependent review of the data and manual tracing at a later time. Very few attempts toward

 automated assessment have been presented [14, 15], all of which have computational requirements that prevent them to be used in real time. As such, they have reduced the appeal for clinical use. In this paper, we describe a method for automated measurement of the abdominal aortic diameter directly from fetal US videos. We propose a neural network architecture that is able to process US videos in real time and leverage both the temporal redundancy of US videos and the quasi-periodicity of the aorta diameter.

The main contributions of the proposed method are as follows. First, we show that a shallow CNN is able to learn imaging features better than two other deep alternatives and outperforms classical methods as level-set for fetal abdominal aorta diameter prediction. Second, we add to the CNN a convolution gated recurrent unit (C-GRU) [16] for exploiting the temporal redundancy of the features extracted by CNN from the US video sequence. Finally, we add a new penalty term to the loss function used to train the CNN to exploit periodic variations.

## Related work

The interest in measuring the diameter and intima-media thickness (IMT) of major vessels has stemmed from its importance as a biomarker of hypertension damage and atherosclerosis in adults. Typically, these vascular biomarkers are assessed on the carotid or brachial arteries by identifying its lumen and the different layers of its wall on high-resolution US images, or the scaling factor that explain its changes [17]. The improvements provided by the design of semi-automatic and automatic methods based mainly on the image intensity profile, distribution and gradients analysis, and more recently on active contours. For a comprehensive review of these classical methods, we refer the reader to [18, 19].

In the prenatal setting, the lower image quality, due to the need of imaging deeper in the mother’s womb and by the movement of the fetus, makes the measurement of vascular biomarkers, although measured on the abdominal aorta, challenging. Methods that proved successful for adult carotid image analysis do not perform well on such data, for which only a handful of methods (semi-automatic or automatic) have been proposed, making use of classical tracing methods and mixture of Gaussian modeling of blood–lumen and media–adventitia interfaces [14], or on level sets segmentation with additional regularizing terms linked to the specific task [15]. However, their sensitivity to the image quality and lengthy computation has prevented its wide adoption in clinical routine.

The solution developed in this work is inspired by recent works reported in the area of deep learning, where CNNs are outperforming classical methods in many medical tasks [20]. The first attempt in using a CNN for the measurement of carotid IMT has been made only recently [21]. The exploitation of temporal redundancy on US sequences was shown to be a solution for improving overall detection results of the fetal heart [22], where a CNN coupled with a recurrent neural network (RNN) is used. The detection of the presence of standard planes from prenatal US data has also been tackled using CNN with long short-term memory (LSTM) [23].

## Datasets

### Real data from pregnancy checks

This study makes use of a dataset consisting of 25 ultrasound video sequences acquired during routine third-trimester pregnancy check-up from the Department of Woman and Child Health of the University Hospital of Padova (Italy). The local ethics committee approved the study and all patients gave written informed consent. The gestational age for the scans we used is 32 weeks and $4\, \mathrm{days} \pm 4\, \mathrm{weeks} \,(\mathrm{mean} \pm \mathrm{stdev})$.

Fetal US data were acquired using a US machine (Voluson E8, GE) equipped with a 5 MHz linear array transducer, according to the guidelines in [24, 25], using a $70^\circ$ FOV, image dimension 720$\times$960 pixels, a variable resolution between 0.03 and 0.1 mm and a mean frame rate of 47 fps. Gain settings were tuned to enhance the visual quality and contrast during the examination. The length of the video is between 2 and 15 s, ensuring that at least one full cardiac cycle is imaged.

After the examination, the video of each patient was reviewed and a relevant video segment was selected for semi-automatic annotation considering its visual quality and length: All frames of the segment were processed with the algorithm described in [14] and then the diameters of all frames in the segments were manually reviewed and corrected. The length of the selected segments varied between 21 frames 0.5 s and 126 frames 2.5 s.

The 25 annotated segments in the dataset were then randomly divided into training ($60\%$ of the segments), validation ($20\%$) and testing ($20\%$) sets. In order to keep the computational and memory requirements low, each frame was cropped to have a square aspect ratio and then resized to $128 \times 128$ pixels. We also make this dataset public to allow for the results to be reproduced (10.6084/m9.figshare.11368019).

**Table 1:** Value of the parameters used to simulate the US sequences

Simulation parameter	Distribution	–	–
$d_0$	Normal	$\mu _d=30$	$\sigma _d=6$
$A_0$	Uniform	$\mathrm{min}_{A}=0.05\cdot d_0$	$\mathrm{max}_{A}=0.35\cdot d_0$
*T*	Normal	$\mu _T=10$	$\sigma _T=3$
$\alpha _0$	Uniform	$\mathrm{min}_\alpha =0$	$\mathrm{max}_\alpha =2\pi$
$\epsilon$	Normal	$\mu _\epsilon =0$	$\sigma _{\epsilon }=0.1$
$\mathrm{imt}_0$	log-Normal	$\mu _{\mathrm{imt}}=0$	$\sigma _{\mathrm{imt}}=0.6$

### Synthetic data

**Fig. 1:**
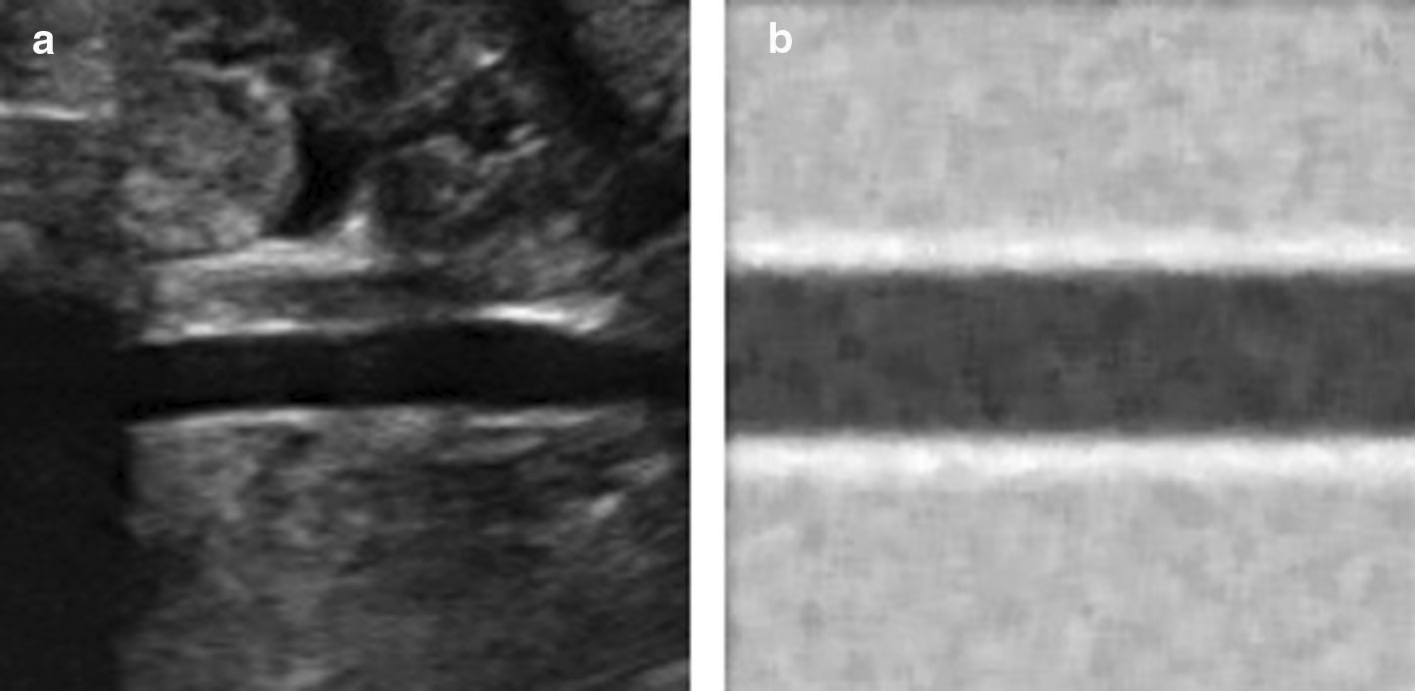
Exemplary real (**a**) and synthetic (**b**) US frames. The synthetic frame presents random Gaussian noise in order to match to the real scenario

A set of 1000 virtual US sequences with 125 frames and corresponding diameter are generated with an in-house software (available at 10.6084/m9.figshare.11368019), trying to capture the relevant appearance of patients’ data without a physics-based simulation. Images of US abdominal aorta are synthesised as illustrated in Fig. 1. They present idealized conditions: a full coverage of the image by the vessel, no confounding structures around the vessel and a sinusoidal movement of the vessel walls.

In order to model the variability to be faced in clinical settings, each US sequence was created by first drawing the vessel lumen with average diameter $d_0\sim {\mathcal {N}}(\mu _d,\sigma _d)$, period $T\sim {\mathcal {N}}(\mu _T,\sigma _T)$, phase $\alpha _{0}\sim {\mathcal {U}}(0,\pi )$ and amplitude $A_0\sim {\mathcal {U}}(A_{\mathrm{min}_{A}},A_{\mathrm{max}_{A}})$. Thus, each US frame in the sequence was created from the diameters *d* calculated:

1 $$\begin{aligned} d = d_{0} + A_{0} \cdot \sin \left( \frac{2\pi \cdot n_{\mathrm{frame}}}{T} + \alpha _{0}\right) + \epsilon \end{aligned}$$

with $\epsilon \sim {\mathcal {N}}(0,\sigma _\epsilon )$ a small random perturbation.

Then, the vessel wall (intima-media thickness) is similarly calculated:

2 $$\begin{aligned} \mathrm{imt} = (3+\mathrm{imt}_{0}) \cdot \left( 1+ 0.2 \cdot \sin \left( \frac{2\pi \cdot n_{\mathrm{frame}}}{T} + \alpha _{0}+\pi \right) \right) \end{aligned}$$

The variation of the vessel has an amplitude of $imt_{0}$, drawn from a log-normal distribution $\mathcal {LN}(\mu _{\mathrm{imt}},\sigma _{\mathrm{imt}})$, and varies sinusoidally in accordance with the diameter, although with a $\pi$ phase shift, so that when the diameter is larger the thickness is smaller (the walls are compressed by the blood pressure within the lumen).

Table 1 summarizes the simulation parameters used. The vessel lumen was assumed to have an average gray-scale value of 0.2, the IMT an average gray scale of 0.8 and the image background of 0.6. A Gaussian noise, with $\mu =5$ and $\sigma ^{2}=2$, and an intensity proportional noise were added to each sequence frame for making the generation the US frames more closely with the real acquisition sequences.

## Network architecture

A solution to predict, from a sequence of ultrasound images, the diameter of the fetal abdominal aorta ${\hat{y}}[t]$ at each time point *t*, without the explicit identification of the lumen or its walls, is described here and available in https://github.com/bionick87/DiameterNet.

**Fig. 2:**
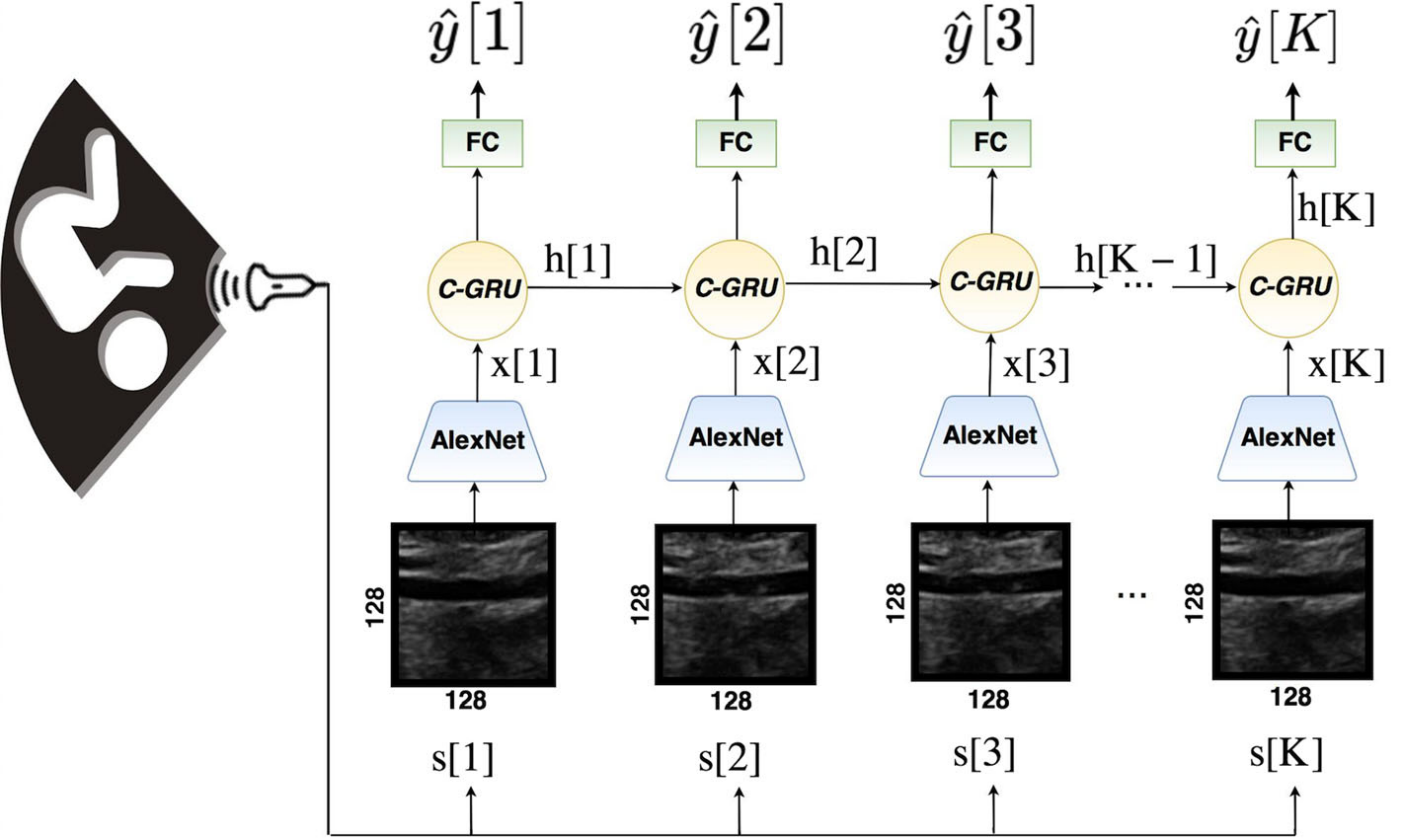
The deep learning architecture proposed for abdominal diameter aorta prediction. The blue blocks represent the CNNs (AlexNet) that extract features *x*[*t*] from each frame *s*[*t*] of the US sequence *S*. Features *x*[*t*] are then passed to Convolution Gated Recurrent Units (C-GRU) (yellow circle) that encode and combine the information from different time points to exploit the temporal coherence. The fully connected block (FC, in green), takes as input the current encoded state *h*[*t*] as feature to estimate the aorta diameter ${\hat{y}}[t]$. Note that the actual implementation is not a set of parallel units per each frame as in the figure, but a single (CNN, C-GRU, FC) chain that is looped through the C-GRU (color figure online)

Our proposed deep learning solution consists of three main components (see Fig. 2): a convolutional neural network (CNN) that captures the salient characteristics from ultrasound input images; a convolution gated recurrent unit (C-GRU) [16] that exploits the temporal coherence through the sequence; and a regularized loss function, called *CyclicLoss*, that better guides the learning through the redundancy between adjacent cardiac cycles.

Our input consists of a set of images of a sequence $S=[s[1], \ldots , s[K]]$ where each image *s*[*t*] has dimension $N \times M$ pixels at time *t*, with $t\in \{1,\ldots ,K\}$. At each time point *t* the CNN extracts the feature maps *x*[*t*] of dimensions $D\times N_x \times M_x$, where *D* is the number of maps, and $N_x$ and $M_x$ are their in-plane pixel dimensions, which depend on the extent of dimensionality reduction obtained by the CNN through its pooling operators.

The feature maps are then processed by a C-GRU layer [16]. The C-GRU combines the current feature maps *x*[*t*] with an encoded representation $h[t-1]$ of the feature maps $\{x[1],\ldots ,x[t-1]\}$ extracted at previous time points of the sequence to obtain an updated encoded representation *h*[*t*], the *current state*, at time *t*: This allows the exploitation of the temporal coherence in the data. The *h*[*t*] of the C-GRU layer is obtained by two- specific gates designed to control the information inside the unit: a reset gate, *r*[*t*], and an update gate, *z*[*t*], defined as follows:

3 $$\begin{aligned} r[t]= \sigma (W_{hr} * h[t-1] + W_{xr}* x[t] + b_{r}) \end{aligned}$$

4 $$\begin{aligned} z[t]= \sigma (W_{hz} * h[t-1] + W_{xz}* x[t] + b_{z}) \end{aligned}$$

Here, $\sigma ()$ is the sigmoid function, $W_{\cdot }$ is recurrent weights matrices whose first subscript letter refers to the input of the convolution operator (either the feature maps *x*[*t*] or the state $h[t-1]$), and whose second subscript letter refers to the gate (reset *r* or update *z*).

All these matrices have a dimension of $D \times 3 \times 3$, and $b_{\cdot }$ is a bias vector. In this notation, $*$ defines the convolution operation. The current state is then obtained as:

5 $$\begin{aligned} h[t] = (1-z[t]) \odot h[t-1] + z[t] \odot \tanh (W_{h}*(r[t] \odot h_{t-1}) + W_{x} *x[t] + b). \end{aligned}$$

Here, $\odot$ denotes the dot product and $W_{h}$ and $W_{x}$ are recurrent weight matrices for $h[t-1]$ and *x*[*t*], used to balance the new information represented by the feature maps *x*[*t*] derived by the current input data *s*[*t*] with the information obtained observing previous data $s[1],\ldots ,s[t-1]$. On the one hand, *h*[*t*] is then passed on for updating the state $h[t+1]$ at the next time point, and on the other is flattened and fed into the last part of the network, built by fully connected (FC) layers progressively reducing the input vector to a scalar output that represents the current diameter estimate ${\hat{y}}[t]$.

### CyclicLoss

Under the assumption that the pulsatility of the aorta follows a periodic pattern with the cardiac cycle, the diameter of the vessel at corresponding instants of the cardiac cycle should ideally be equal. Assuming a known cardiac period $T_{\mathrm{period}}$, we propose to add a regularization term to the loss function used to train the network that penalizes large differences of the diameter values that are estimated at time points that are one cardiac period apart.

We call this regularization term *CyclicLoss* (CL), computed as $L_2$ norm of the difference between pairs of predictions at the same point of the heart cycle and from adjacent cycles:

6 $$\begin{aligned} \mathrm{CL}=\sqrt{\sum _{n=0}^{N_{\mathrm{cycles}}-1} \sum _{t=0}^{T_{\mathrm{period}}} \left( {\hat{y}}[t+(n+1)T_{\mathrm{period}}] - {\hat{y}}[t+nT_{\mathrm{period}}]\right) ^2} \end{aligned}$$

The $T_{\mathrm{period}}$ is the period of the cardiac cycle, while $N_{\mathrm{cycles}}$ is the number of integer cycles present in the sequence and ${\hat{y}}[t]$ is the estimated diameter at time *t*. $T_{\mathrm{period}}$ is determined by a peak detection algorithm on the training data *y*[*t*], and the average of all peak-to-peak detection distances define its value. Accordingly, $N_{\mathrm{cycles}}$ is the number of cycles present, calculated as the total length of the *y*[*t*] signal divided by $T_{\mathrm{period}}$.

The loss to be minimized is therefore a combination of the classical mean squared error (MSE) with the CL, and the balance between the two is controlled by a constant $\lambda$:

7 $$\begin{aligned} \mathrm{Loss} = \mathrm{MSE} + \lambda \cdot \mathrm{CL} = \frac{1}{K} \sum _{t=1}^{K} (y[t]-{\hat{y}}[t])^{2} + \lambda \cdot \mathrm{CL}, \end{aligned}$$

where *y*[*t*] is the target diameter at time point *t*.

It is worth noting that the knowledge of the period of the cardiac cycle is needed only during the training phase when the loss is being minimized. During the test phase, on an unknown image sequence, the trained network provides its estimate blind of the periodicity of the specific sequence under analysis.

### Implementation details

For our experiments, we chose AlexNet [26] as a feature extractor for its simplicity and its better performance as compared to other deeper CNNs (see Results section). It has five hidden layers with $11 \times 11$ kernel size in the first layer, $5 \times 5$ in the second and $3 \times 3$ in the last three layers; it is well suited to the low image contrast and diffuse edges characteristic of US sequences. Each network input for the training is a sequence of $K=[25,125]$ ultrasound frames with $N=M=128$ pixels, AlexNet provides feature maps of dimension $D\times N \times M=256\times 13 \times 13$, and the final output ${\hat{y}}[t]$ is the estimated abdominal aorta diameter value at each frame.

The loss function is optimized with the Adam algorithm [27], which is a first-order gradient-based technique. The learning rate used is $1e^{-4}$ with the iterations calculated as a number of patients for training $\times$ number of ultrasound frames for 100 epochs. In order to improve generalization, data augmentation of the input with a vertical and horizontal random flip is used at each iteration. The best cross-validated $\lambda$ constant, used during training with *CyclicLoss*, takes the value of $1e^{-6}$.

## Experiments

### Architecture design and comparison to state-of-the-art

The proposed solution is tested in the real datasets to evaluate the different architectural choices. In order to understand the behavior of different features extraction methods, we explored the performance of deeper network architectures, and so AlexNet was replaced by InceptionV4 [28] and DenseNets 121 [29]. The addition of both the recurrence mechanism and the cyclic loss are also tested, and all possibilities are benchmarked against a state-of-the-art method that uses traditional image analysis concepts. The specific choice for this method is the one that is reported to be the best for the challenging task of the fetal aorta, and that is based on level sets [15].

**Fig. 3:**
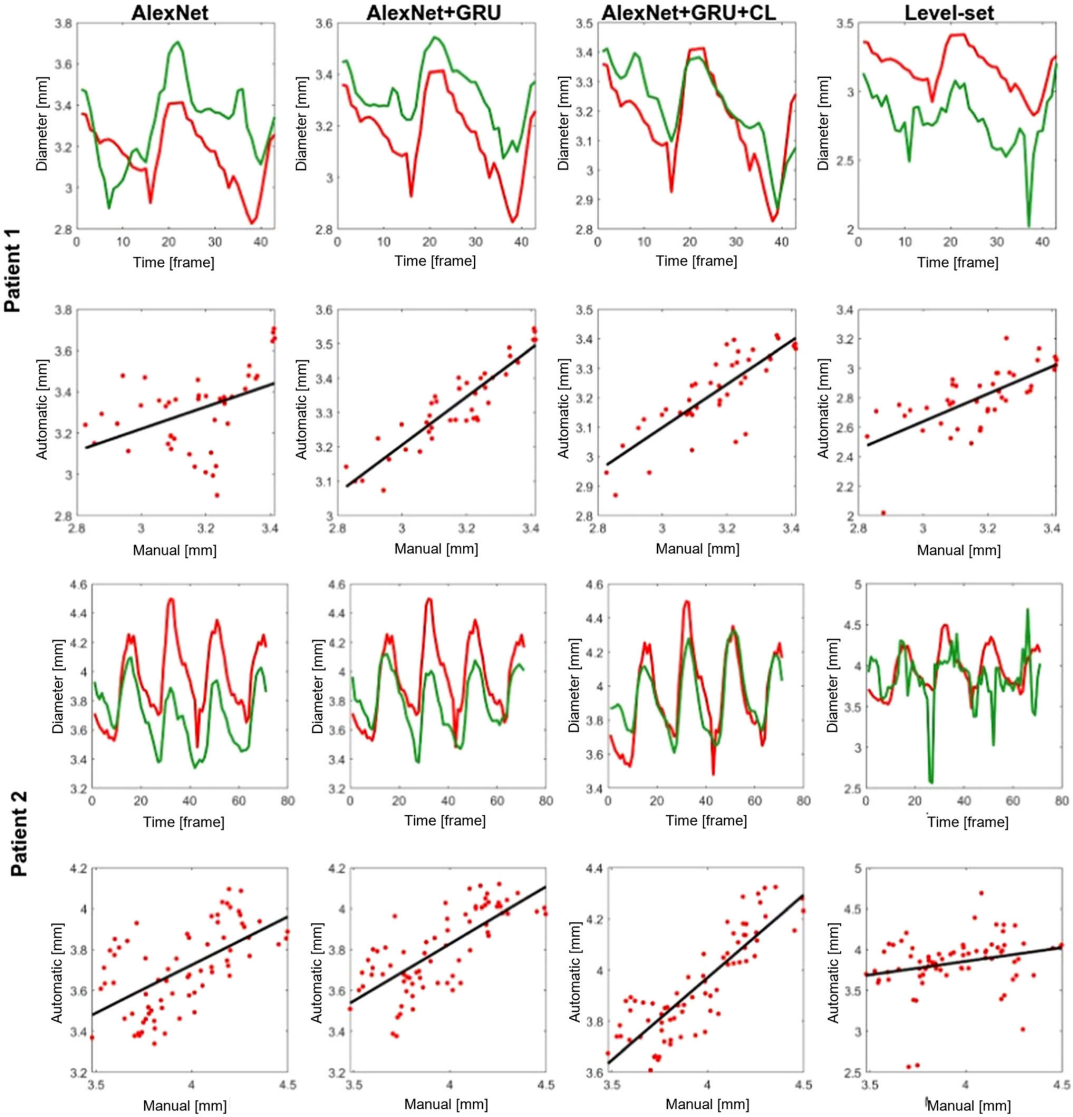
Estimation of the fetal aortic diameter from the real test set data in two exemplary cases, using four different methods: AlexNet, AlexNet+C-GRU, AlexNet+C-GRU trained with the *CyclicLoss* and level set method. Two plots are reported for each case and method: (top) the temporal transients of changes in diameter (ground truth in red line) and (bottom) the regression plot between manual and automatic methods (color figure online)

The performance of each method was evaluated both with respect to the mean squared error (MSE) and to the mean absolute relative error (RE); all values are reported in Table 2 in terms of average and standard deviation across the test set. In order to provide a visual assessment of the performance, representative estimations on two sequences of the test set are shown in Fig. 3. Further, a non-parametric test (Kolmogorov–Smirnov) was performed to check if the best model was statistically different compared to the others. The results obtained with the complete model AlexNet+C-GRU+CL are better and significantly different from all others (p 0.05). It is also worth noting that the use of C-GRU greatly improves the performance of all CNNs, dense or shallow, both in terms of MSE and of RE. Finally, the statistical number of test and validation samples, in the synthetic dataset, are adequate for a correct validation, then no cross-validation is necessary.

**Table 2:** Performance results in the real dataset: mean squared error (MSE) and relative error (RE) for all methods—error values are average (standard deviation)

Methods	MSE ($\mathrm{mm}^2$)	RE (%)	*p*-value
AlexNet	0.29 (0.09)	8.67 (10)	1.01e-12
AlexNet+C-GRU	0.093 (0.191)	6.11 (5.22)	1.21e-05
**AlexNet+C-GRU+CL**	**0.085 (0.17)**	**5.23 (4.91)**	“–”
DenseNet121	0.31 (0.56)	9.55 (8.52)	6.00e-13
DenseNet121+C-GRU	0.13 (0.21)	7.72 (5.46)	7.78e-12
InceptionV4	6.81 (14)	50.4 (39.5)	6.81e-12
InceptionV4+C-GRU	0.76 (1.08)	16.3 (9.83)	2.89e-48
Level-set	0.31 (0.80)	8.13 (9.39)	1.9e-04

AlexNet+C-GRU+CL yields the best performance

### Testing the limitation of the lack of training data

Strikingly, we observed that deep CNNs are not able to outperform AlexNet on the dataset that was available for this work. One possible explanation was the lack of enough training data. The synthetic cohort is thus used to investigate if an unlimited source of imaging data could guide deeper CNNs to learn the required robust features.

The training and testing are conducted in the same manner as with the real data, and CNNs were enriched with both *CyclicLoss* and C-GRU. Results are consistent with the previous: as shown in Table 3, the shallow network AlexNet outperforms the two deeper choices. Specifically, DenseNet121 diverged during training, and InceptionV4 achieved a reasonable convergence but with less accuracy than AlexNet.

**Fig. 4:**
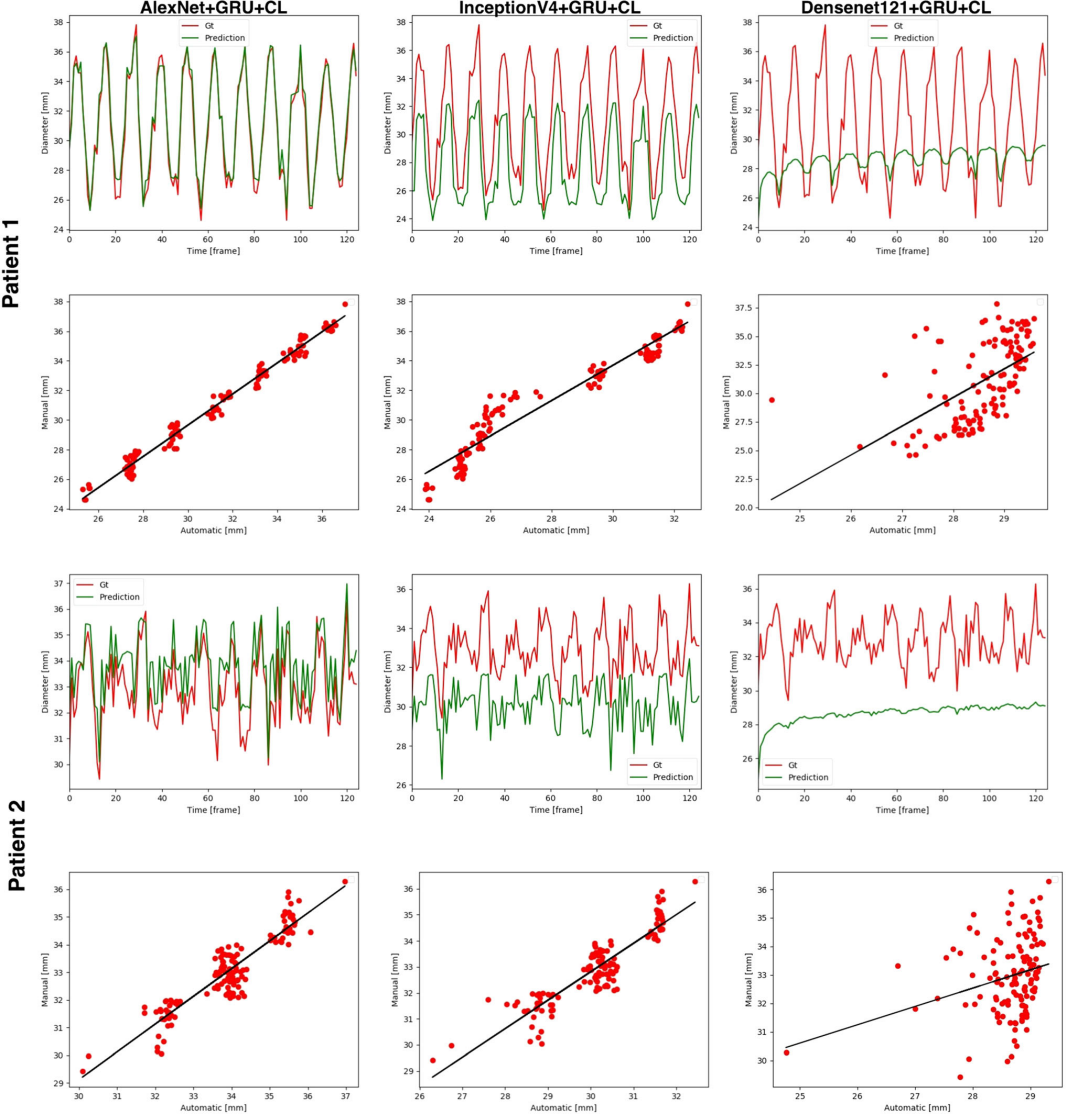
Example of diameter prediction on synthetic data for the three CNNs for two different patients. The shallow CNN, AlexNet, shows a good agreement with the ground truth signal (red line) while deeper CNNs (InceptionV4 and Densenet121) show a loss of precision. The underlying linear regression plot shows the presence of clusters, in a variable number. The smaller the number of these clusters, the larger the empty space between them and the larger the loss of precision during the process of inference by the recurrent unit (color figure online)

The regression line between predictions and ground truth reveals that estimated values are clustered around the regression line (see Fig. 4), no matter if a shallow or dense CNN is used. The gain in accuracy is thus linked to the existence of a larger number of these clusters, where the limit they will disappear when the predicted signal is equal to the ground truth. It is verified that this pattern is only present with the synthetic and not with the real data (compare to Fig. 3).

**Table 3:** Performance results in the synthetic dataset: mean squared error (MSE), relative error (RE), mean absolute error (MAE) and coefficient of determination ($R^2$) for the three methods compared, expressed as average (standard deviation)

Methods	MSE ($\mathrm{pixel}^2$)	RE (%)	MAE ($\mathrm{pixel}$)	$R^2$
**AlexNet+C-GRU+CL**	**0.02 (0.02)**	**1.96 (0.80)**	**0.58 (0.36)**	**0.92 (0.10)**
DenseNet121+C-GRU+CL	0.04 (0.18)	12.81 (7.76)	3.95 (2.89)	0.32 (0.25)
InceptionV4+C-GRU+CL	3.38 (2.21)	10.90 (3.90)	3.36 (1.51)	0.79 (0.14)

Bold values indicate the method that achieved the best performance

## Discussion and conclusion

The deep learning (DL) architecture proposed shows excellent performance compared to traditional image analysis methods, both in accuracy and efficiency. This improvement is achieved through a combination of a shallow CNN and the exploitation of the temporal and cyclic coherence. Our results indicate that a shallow CNNs performs better than deeper CNNs such as DenseNet 121 and InceptionV4 and that this is not caused by the lack of training data.

### The *CyclicLoss* benefits

The exploitation of temporal coherence is what pushes the performance of the DL solution beyond current image analysis methods, reducing the MSE from $0.29\,\mathrm{mm}^2$ (naive architecture) to $0.09\,\mathrm{mm}^2$ with the addition of the C-GRU. The *CyclicLoss* is an efficient way to better guide the training of the DL solution in case of data showing some periodicity, as in cardiovascular imaging. Please note that the knowledge of the signal period is only required by the network during training, and as such it does not bring additional requirements on the input data for real clinical application. We interpret that the *CyclicLoss* is making the network to better extract the useful ultrasound features, and to learn to expect a periodic input and provide some periodicity in the output sequence.

### The depth required in the CNN to analyze ultrasound sequences

Results in both the real and the synthetic datasets show that the sallow CNN outperforms two alternative deep CNNs. The synthetic dataset has a very simple set of features (i.e., two horizontal lines) without any confounding structure in the images. An ultrasound image is not as rich in features as a picture of a cat that is used in the design of deep CNNs such as InceptionV4 and Densenet121, and this may be the reason why the shallow CNN has outperformed the other two. Further experimentation is needed in order to generalize these findings.

One surprising finding was the presence of a clustered regression line between predictions and ground truth values of diameter is shown in Fig. 4. The fact that this originates from the solution regardless of the depth of the CNN does suggest that is caused by the regression unit and might be a limitation of the use of the *CyclicLoss* for training. The other hint was the occurrence of this phenomena only with synthetic data, not with the real sequences, which indicates that this behavior originates from some feature of the idealized images. In any case, our choice for a C-GRU was motivated by two particular advantages compared to previous approaches [22, 23]: first, it is not subject to the vanishing gradient problem like the RNN, allowing the training from long sequences of data. And second, it has less computational cost compared to the LSTM, and that makes it suitable for real-time video application.

### Limitations and future works

This work assumes the presence of the vessel in the current field of view, and thus requires a preliminary solution to identify it will be required and may decrease the performance and throughput. Further research is thus required to evaluate how well the solution adapts to the scenario of lack of cyclic consistency during training, or when the vessel of interest can move in and out of the field of view during the acquisition, or to investigate the possibility of a concurrent estimation of the cardiac cycle and vessel diameter.

## References

[1] Visentin S, Grumolato F, Nardelli GB, Di Camillo B, Grisan E, Cosmi E (2014). Early origins of adult disease: low birth weight and vascular remodeling. Atherosclerosis.

[2] Lewandowski AJ, Augustine D, Lamata P, Davis EF, Lazdam M, Francis J, McCormick K, Wilkinson AR, Singhal A, Lucas A, Smith NP, Neubauer S, Leeson P (2013). Preterm heart in adult life: cardiovascular magnetic resonance reveals distinct differences in left ventricular mass, geometry, and function. Circulation.

[3] Skilton MR, Evans N, Griffiths KA (2005). Aortic wall thickness in newborns with intrauterine growth restriction. Lancet.

[4] Sookoian S, Gianotti TF, Burgueño AL, Pirola CJ (2013). Fetal metabolic programming and epigenetic modifications: a systems biology approach. Pediatr Res.

[5] Crispi F, Miranda J, Gratacós E (2017). Long-term cardiovascular consequences of fetal growth restriction: biology, clinical implications, and opportunities for prevention of adult disease. Am J Obstet Gynecol.

[6] Visentin S, Londero AP, Calanducci M, Grisan E, Bongiorno MC, Marin L, Cosmi E (2019). Fetal abdominal aorta: doppler and structural evaluation of endothelial function in intrauterine growth restriction and controls. Ultraschall Med.

[7] Jarvisalo MJ, Jartti L, Nanto-Salonen K (2001). Increased aortic intima media thickness: a marker of preclinical atherosclerosis in high-risk children. Circulation.

[8] Koklu E, Ozturk MA, Gunes T, Akcakus M, Kurtoglu S (2007). Is increased intima media thickness associated with preatherosclerotic changes in intrauterine growth restricted newborns?. Acta Paediatr.

[9] Gomez-Roig MD, Mazarico E, Valladares E, Guirado L, Fernandez-Arias M, Vela A (2015). Aortic intima-media thickness and aortic diameter in small for gestational age and growth restricted fetuses. PLOS One.

[10] Rafferty AR, D’Arcy C, Cann L, Pyman J, Rogers P, Davis PG, Nowell C, Burgner D (2017). Histological changes in the umbilical artery following severe chorioamnionitis and funisitis may be indicative of early atherosclerosis. Placenta.

[11] Di Bernardo S, Mivelaz Y, Epure AM, Vial Y, Simeoni U, Bovet P, Estoppey Younes S, Chiolero A, Sekarski N (2017). MySweetHeart research group: assessing the consequences of gestational diabetes mellitus on offspring’s cardiovascular health: MySweetHeart cohort study protocol, Switzerland. BMJ Open.

[12] Valenzuela-Alcaraz B, Serafini A, Sepulveda-Martínez A, Casals G, Rodríguez-López M, Garcia-Otero L, Cruz-Lemini M, Bijnens B, Sitges M, Balasch J, Gratacós E, Crispi F (2019). Postnatal persistence of fetal cardiovascular remodelling associated with assisted reproductive technologies: a cohort study. BJOG.

[13] Lo Vasco VR, Salmaso R, Zanardo V, Businaro R, Visentin S, Trevisanuto D, Cosmi E (2011). Fetal aorta wall inflammation in ultrasound-detected aortic intima/media thickness and growth retardation. J Reprod Immunol.

[14] Veronese E, Tarroni G, Visentin S, Cosmi E, Linguraru MG, Grisan E (2014). Estimation of prenatal aorta intima-media thickness from ultrasound examination. Phys Med Biol.

[15] Tarroni G, Visentin S, Cosmi E, Grisan E (2015) Fully-automated identification and segmentation of aortic lumen from fetal ultrasound images. In: IEEE EMBC, pp 153–156 10.1109/EMBC.2015.7318323 26736223

[16] Siam M, Valipour A, Jägersand M, Ray N (2017) Convolutional gated recurrent networks for video segmentation. In: IEEE ICIP conference, pp 3090–3094

[17] Frangi A, Laclaustra M, Lamata P (2003). A registration-based approach to quantify flow-mediated dilation (FMD) of the brachial artery in ultrasound image sequences. IEEE Trans Med Imag.

[18] Molinari F, Zeng G, Suri JS (2010). A state of the art review on intima-media thickness (IMT) measurement and wall segmentation techniques for carotid ultrasound. Comp Meth Prog Biomed.

[19] Loizou CP (2014). A review of ultrasound common carotid artery image and video segmentation techniques. Med Biol Eng Comp.

[20] Litjens G, Kooi T, Bejnordi BE, Setio AAA, Ciompi F, Ghafoorian M, van der Laak JAWM, van Ginneken B, Sánchez CI (2017). A survey on deep learning in medical image analysis. Med Image Anal.

[21] Shin JY, Tajbakhsh N, Hurst RT, Kendall CB, Liang J (2016) Automating carotid intima-media thickness video interpretation with convolutional neural networks. In: IEEE CVPR conference, pp 2526–2535

[22] Huang Weilin, Bridge Christopher P., Noble J. Alison, Zisserman Andrew (2017). Temporal HeartNet: Towards Human-Level Automatic Analysis of Fetal Cardiac Screening Video. Lecture Notes in Computer Science.

[23] Chen Hao, Dou Qi, Ni Dong, Cheng Jie-Zhi, Qin Jing, Li Shengli, Heng Pheng-Ann (2015). Automatic Fetal Ultrasound Standard Plane Detection Using Knowledge Transferred Recurrent Neural Networks. Lecture Notes in Computer Science.

[24] Cosmi E, Visentin S, Fanelli T, Mautone AJ, Zanardo V (2009). Aortic intima-media thickness in fetuses and children with intrauterine growth restriction. Obs Gyn.

[25] Skilton MR, Evans N, Griffiths KA, Harmer JA, Celermajer DS (2005). Aortic wall thickness in newborns with intrauterine growth restriction. Lancet.

[26] Krizhevsky A, Sutskever I, Hinton GE (2012) ImageNet classification with deep convolutional neural networks. In: NIPS-2012, pp 1097–1105

[27] Kingma DP, Ba LJ (2015) Adam: a method for stochastic optimization. In: 3rd international conference for learning representations

[28] Szegedy C, Ioffe S, Vanhoucke V (2017) Inception-v4, inception-ResNet and the impact of residual connections on learning. In: AAAI-17, pp 4278–4284

[29] Huang G, Liu Z, van der Maaten L, Weinberger KQ (2017) Densely connected convolutional networks. In: IEEE CVPR Conference, pp 2261–2269

